# Incidence and Clinical Characteristics of Respiratory Sarcopenia in Community-Dwelling Older Adults: A Cross-Sectional Study

**DOI:** 10.7759/cureus.90304

**Published:** 2025-08-17

**Authors:** Urara Chiba, Tomoyuki Morisawa, Yota Kunieda, Shoki Yusu, Kohei Kawamura, Tomokazu Takakura, Masakazu Saitoh, Kotaro Iwatsu, Tetsuya Takahashi, Toshiyuki Fujiwara

**Affiliations:** 1 Department of Physical Therapy, Graduate School of Health Science, Juntendo University, Tokyo, JPN; 2 Department of Rehabilitation Medicine, Juntendo Tokyo Koto Geriatric Medical Center, Tokyo, JPN; 3 Department of Physical Therapy, Faculty of Health Science, Juntendo University, Tokyo, JPN; 4 Department of Rehabilitation Medicine, Juntendo University Graduate School of Medicine, Tokyo, JPN; 5 Department of Rehabilitation Medicine, Juntendo University School of Medicine, Tokyo, JPN

**Keywords:** cognitive function, community-dwelling older adults, oral function, respiratory muscle, sarcopenia

## Abstract

Purpose: This study aimed to determine the prevalence, characteristics, and associated factors of respiratory sarcopenia in community-dwelling older adults.

Methods: This cross-sectional study included 369 community-dwelling older adults aged 65 years and above, living in Koto-ku, Tokyo, Japan. Measurements included body composition, physical function (grip strength, walking speed, and 5 chair-stand, 5CS, test), physical activity (the International Physical Activity Questionnaire-Short Form), respiratory muscle strength (%Maximal Inspiratory Pressure, %MIP, and %Maximal Expiratory Pressure, %MEP), oral function (oral diadochokinesis, ODK; tongue pressure), cognitive function (Montreal Cognitive Assessment-Japanese), and health-related quality of life (the MOS 8-Item Short-Form Health Survey). Participants were classified into three mutually exclusive groups based on respiratory muscle strength and limb skeletal muscle mass. The Probable respiratory sarcopenia group (Probable group) included individuals with both reduced respiratory muscle strength (defined as both %MIP and %MEP values below 80%) and low limb skeletal muscle mass (defined as <7 kg/m² in men and <5.7 kg/m² in women, based on the Asian Working Group of Sarcopenia 2019). The Possible respiratory sarcopenia group (Possible group) included participants with reduced respiratory muscle strength alone but normal skeletal muscle mass. The Robust group comprised those with neither respiratory muscle weakness nor low skeletal muscle mass. The prevalence of each group was calculated separately. For further analysis, the Probable and Possible groups were combined into a single respiratory sarcopenia group, and the Robust group was used as the control. A t-test, Mann-Whitney U test, and chi-square test were used to compare the characteristics of each group. Logistic regression analysis was then performed to identify factors associated with the presence of respiratory sarcopenia.

Results: The prevalence of Probable respiratory sarcopenia was 3.3%, and that of Possible respiratory sarcopenia was 33.3%. Compared with the Robust group, the respiratory sarcopenia group exhibited significantly poorer physical function, including grip strength, gait speed, 5CS performance, physical activity, and oral function. Additionally, cognitive function was significantly lower in the respiratory sarcopenia group. Significant factors associated with respiratory sarcopenia included grip strength, 5CS, moderate physical activity, tongue pressure, and fat-free mass.

Conclusion: The prevalence of Probable respiratory sarcopenia and Possible respiratory sarcopenia among community-dwelling older adults was 3.3% and 33.3%, respectively. Respiratory sarcopenia was characterized by significantly lower physical function (grip strength, gait speed, and 5CS), physical activity, oral function (ODK and tongue pressure), and cognitive function. Furthermore, grip strength, 5CS score, moderate physical activity, and tongue pressure were identified as significant factors associated with respiratory sarcopenia, suggesting that it requires a comprehensive evaluation including physical function, physical activity, oral function, and cognitive function.

## Introduction

Respiratory sarcopenia is a condition where both respiratory muscle weakness and muscle mass decrease [1]. Patients with respiratory sarcopenia are more likely to have decreased activities of daily living (ADL) [2] and higher mortality rates [3]. Since respiratory sarcopenia has a poor prognostic factor, early screening is important. The prevalence of respiratory muscle weakness among community-dwelling older adults is 54.7%, and the prevalence of both generalized sarcopenia and respiratory muscle weakness is 12% in Japan [4]. However, no unified diagnosis of respiratory sarcopenia currently exists, and the criteria used in different studies differ, making it difficult to determine respiratory sarcopenia prevalence. In 2021, the Japanese Society of Rehabilitation and Nutrition proposed diagnostic criteria for respiratory sarcopenia [5]. In 2023, a new set of diagnostic criteria was published in a joint position paper by four societies [1]. This position paper is a compilation of previous studies [4,6] on respiratory sarcopenia. Clarifying the prevalence and characteristics of respiratory sarcopenia using these diagnostic criteria is essential.

Older patients with respiratory sarcopenia have significantly lower grip strength, knee extension muscle strength, normal walking speed, 5 chair-stand (5CS) test results, and one-leg standing time [4]. Low nutrition reportedly contributes to muscle weakness [7]; it is caused not only by respiratory muscle weakness but also by oral frailty [8]. Oral frailty is a state of oral function between the normal state of a healthy mouth and the decline of oral function, causing sarcopenia, frailty, and undernutrition [9]. These findings suggest that age-related physiological changes in older adults, such as decreased physical and oral function and reduced nutritional status, may interact with each other and influence the development of respiratory sarcopenia. Therefore, it is important to examine the relationship between respiratory sarcopenia, oral function, and nutrition that has not been previously reported. It is hypothesized that factors related to respiratory sarcopenia involve both physical function and multiple factors, such as oral function, cognitive function, and quality of life. However, no previous reports exist on comprehensive investigations regarding factors specific to older adults.

Respiratory sarcopenia, common in community-dwelling older adults, can be related to limb skeletal muscle strength and physical function based on the mechanism of the respiratory metabolic reflex [10]. As sarcopenia is often caused by poor oral function, low nutrition, and low physical activity, we hypothesized that sarcopenia is related to respiratory sarcopenia.

This study aimed to examine the prevalence and characteristics of respiratory sarcopenia and its associated factors in community-dwelling older adults, using the diagnostic criteria from a new position paper published by four professional organizations.

## Materials and methods

Design and participants

This observational cross-sectional study included 369 adults, aged 65 years or older, who attended physical and cognitive function measurement sessions at three welfare centers for older adults in Koto-ku, Tokyo, Japan, between March 2021 and January 2022. As this study was conducted in collaboration with the three facilities mentioned above, data collection was carried out at these facilities. Participants were recruited through posters on the website and at each older adult’s welfare center, and through publicity in each community, and were randomly selected by lottery from among the older adults who consented to participate in the measurement sessions. The inclusion criteria were ADL independence and consent to participate in the study. The exclusion criteria were a history of respiratory disease or treatment for respiratory disease, those with a diagnosis of dementia, intravital implantable electronic devices, metal implants in the body owing to joint replacement or spinal fusion surgery, and missing data. The study was verbally explained to the participants, and written informed consent was obtained before the study was conducted. This study was approved by Juntendo University Tokyo Koto Geriatric Medical Center Ethics Committee (approval number: G20-0016).

Measurement items

Basic Information

Basic information was obtained using a self-administered questionnaire regarding age, sex, educational history (years of education since primary school), and medical history (cancer, heart disease, stroke, hypertension, diabetes, dyslipidemia, fractures, trauma from falls, osteoporosis, arthritis, dementia, and respiratory disease).

Respiratory Muscle Strength

Maximal inspiratory pressure (MIP) and maximal expiratory pressure (MEP) were used to measure respiratory muscle strength using a respiratory muscle strength-measuring device (IOP-01; Kibata Keiki Seisakusho Co., Ltd., Osaka, Japan). Participants performed two maximal inspiratory efforts and two maximal expiratory efforts in the sitting position. The highest values for each were recorded as the measured MIP and MEP. Predicted MIP and MEP values were adjusted for age, height, weight, and sex according to the method by Suzuki et al. [11] (male MIP: 45 - 0.74 age + 0.27 height + 0.60 weight; male MEP: 25.1 - 0.37 age + 0.20 height + 1.20 weight; female MIP: -1.5 - 0.41 age + 0.48 height + 0.12 weight; and female MEP: -19.1 - 0.18 age + 0.43 height + 0.56 weight). Measured MIP and MEP values were subsequently divided by their respective predicted values to obtain %MIP and %MEP.

Physical Function, Frailty, Sarcopenia, and Body Composition

Physical function was measured using grip strength, 6 m comfort walking speed, and 5CS. These are commonly used indicators for screening in older adults. Grip strength was measured bilaterally in the standing and elbow extension positions using a Smedley digital grip strength meter (Grip-D; Takei Kiki Kogyo, Niigata, Japan), and the maximum value was recorded. The comfortable walking speed was measured once at the speed of a normal street walker for a straight line of 10 m (including 2 m each for acceleration and deceleration). The 6 m comfortable walking time was converted to speed (m/s). 5CS was measured by repeatedly standing up five times from a 40 cm high chair with arms crossed in front of the chest, measured using a stopwatch. Frailty was assessed using the Japanese version of the Cardiovascular Health Study criteria [12], with Robust, prefrail, and frail statuses determined based on grip strength, walking speed, physical activity level, and nutritional status. Sarcopenia was determined using the Asian Working Group of Sarcopenia 2019 (AWGS 2019) [13]. Frailty and sarcopenia were categorized after determination and analysis. Height and weight were measured, and body mass index (BMI) was calculated as weight (kg) divided by height squared (m^2^). Body composition was measured using a multifrequency body composition analyzer (MC-780A-N, Tanita, Tokyo, Japan) to determine skeletal muscle mass index, fat-free mass (FFM), percent body fat, and phase angle.

Physical Activity

Physical activity was assessed using the International Physical Activity Questionnaire (IPAQ) Short Form, comprising four questions and scoring three types of physical activity (walking, moderate physical activity, and strong physical activity), per the IPAQ guidelines [14]. Total physical activity was determined by summing the MET minutes/week across all three categories. Participants were then categorized into low, moderate, and high physical activity groups based on their total MET minutes/week.

Oral Function

Oral function was assessed using oral diadochokinesis (ODK) and tongue pressure, measured using an oral function measuring device (TKK 3351 Digital Counter; Takei Kiki Kogyo, Niigata, Japan). The participants were instructed to repeat each syllable, (Pa), (Ta), (Ka), as quickly as Possible for five seconds, and the number of repetitions was counted using a digital counter. The number of pronunciations per second for each syllable was calculated separately. Tongue pressure was measured using a tongue pressure-measuring device (JMS Tongue Pressure Meter TPM02, JMS Corporation, Hiroshima, Japan). The participants were instructed to hold the tongue pressure probe between their tongue and palate, raise the tongue, and press the balloon as hard as Possible against the palate [15]. Tongue pressure was recorded thrice, and the maximum of the three measurements was recorded.

Cognitive Function

Cognitive function was assessed using the Japanese version of the Montreal Cognitive Assessment-Japanese [16], which assesses visuospatial function, executive function, naming, memory, attention, recitation, word recall, abstract concepts, delayed replay, and registration with mild cognitive impairment.

Health-Related Quality of Life

The MOS 8-Item Short-Form Health Survey (SF-8) [17] was used to assess health-related quality of life. SF-8 is a globally widespread assessment of health-related quality of life, measuring eight dimensions of health. Physical and mental summary scores were calculated based on the eight health concepts.

Definition and grouping of respiratory sarcopenia

Participants were classified into three mutually exclusive groups based on respiratory muscle strength and limb skeletal muscle mass, according to the respiratory sarcopenia statement [1]. The Probable respiratory sarcopenia group (Probable group) included individuals with both reduced respiratory muscle strength (defined as both %MIP and %MEP <80%) [18] and low limb skeletal muscle mass (defined as <7 kg/m² for men and <5.7 kg/m² for women, based on AWGS 2019 criteria) [13]. The Possible respiratory sarcopenia group (Possible group) included those with reduced respiratory muscle strength alone but normal limb skeletal muscle mass. The Robust group comprised participants with neither respiratory muscle weakness nor low skeletal muscle mass. The prevalence of each group was calculated separately. For further analysis, the Probable and Possible groups were combined into a single respiratory sarcopenia group, and the Robust group served as the control.

Statistical analysis

Data are presented as mean ± standard deviation, with median and interquartile range used where normal distribution was not shown. Comparisons were made using independent two-group t-tests for continuous variables of basic information, percentage of frailty/sarcopenia, body composition, physical function, physical activity, respiratory muscle strength, oral function, cognitive function, and health-related quality of life in the Robust and respiratory sarcopenia groups. The Mann-Whitney U test was used to compare nonnormally distributed variables, while the chi-square test was used for categorical variables. Before conducting logistic regression analysis, multicollinearity was assessed using variance inflation factors (VIFs). Variables with VIFs greater than 10 were excluded from the model [19]. Logistic regression analysis was then performed using the forced entry method to identify factors associated with respiratory sarcopenia. Independent variables for the logistic regression model were selected based on the results of univariate analyses and previous literature. The statistical significance level was set at 5%, and all analyses were performed using IBM Statistical Package for the Social Sciences Statistics for Windows (version 21.0; IBM Corp., Armonk, NY).

## Results

Attributes of the participants

Of the initially recruited 423 participants, 34 were excluded owing to a history of respiratory disease and 20 owing to missing data, resulting in a final sample size of 369 participants. Basic participants’ information is presented in Table 1. The mean age was 77.1 ± 5.8 years, with 84.6% being female participants; BMI, 22.1 ± 3.3 kg/m^2^; education, 12.9 ± 2.5 years; and the prevalence of frailty and sarcopenia, 19.7% (72 participants) and 4.6% (17 participants), respectively.

**Table 1:** Basic information about the participants SD, standard deviation; BMI, body mass index

Parameter	Value	Range
Age, year, mean ± SD	77.1 ± 5.8	65-94
Sex (female), n (%)	312 (84.6)	-
BMI, kg/m^2^, mean ± SD	22.1 ± 3.3	15-34
Education history, year, mean ± SD	12.9 ± 2.5	6-26
Medical history, n (%)		
Cancer	44 (11.9)	-
Heart disease	33 (8.9)	-
Stroke	12 (3.3)	-
Hypertension	116 (31.4)	-
Diabetes	25 (6.8)	-
Dyslipidemia	42 (11.4)	-
Fracture	10 (2.7)	-
Osteoporosis	57 (15.4)	-
Osteoarthritis	66 (17.9)	-
Dementia	0 (0)	-
Frailty, n (%)	72 (19.7)	-
Sarcopenia, n (%)	17 (4.6)	-

Prevalence and characteristics of respiratory sarcopenia

**Figure 1:**
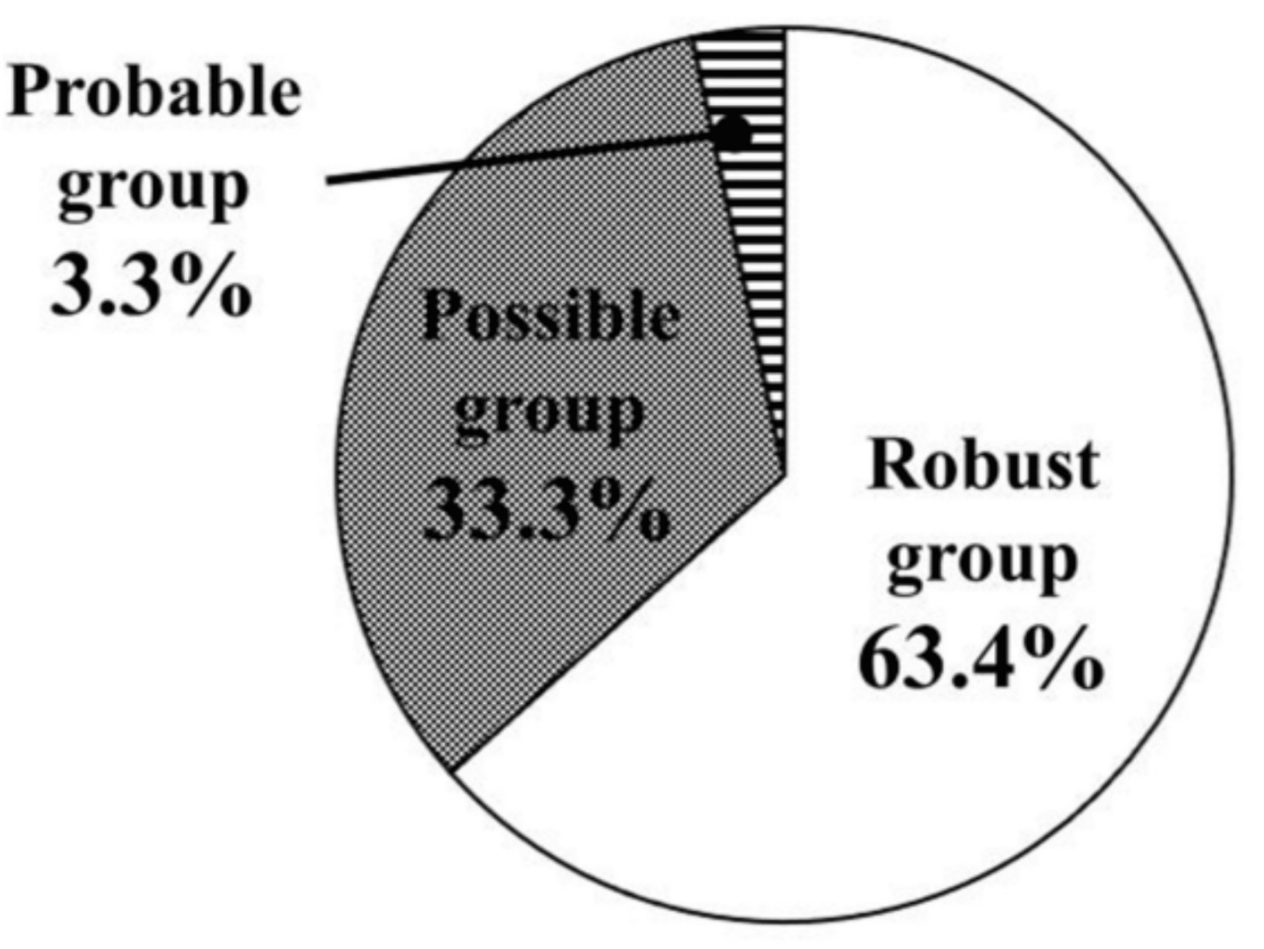
Prevalence of respiratory sarcopenia

Of the 369 participants, 63.4% (234 participants) belonged to the Robust group, 33.3% (123 participants) to the Possible group, and 3.3% (12 participants) to the Probable group (Figure 1). The results of the comparison of the basic information, percentage of frailty/sarcopenia, body composition, physical function, physical activity, respiratory muscle strength, oral function, cognitive function, and health-related quality of life between the Robust and Respiratory Sarcopenia groups are presented in Table 2. Compared to the Robust group, the respiratory sarcopenia group showed significantly reduced physical function, including lower grip strength and slower walking speed, along with poorer 5CS performance (i.e., higher 5CS score). In addition, individuals in the respiratory sarcopenia group demonstrated lower levels of physical activity, as well as significant declines in respiratory muscle strength, oral function, and cognitive function.

**Table 2:** Comparison of the groups ^*^p value of <0.05 (Mann-Whitney U test) ^†^p value of <0.05 (chi-square test) was considered statistically significant SD, standard deviation; IQR, interquartile range; BMI, body mass index; BIA, bioelectrical impedance analysis; SMI, skeletal muscle index; BF, body fat; FFM, fat-free mass; PBF, percent body fat; PhA, phase angle; IPAQ, International Physical Activity Questionnaire; MIP, maximal inspiratory pressure; MEP, maximal expiratory pressure; ODK, oral diadochokinesis; TP, tongue pressure; MoCA-J, Montreal Cognitive Assessment-Japanese; QOL: quality of life; SF-8, short form 8; PCS, physical component summary; MCS, mental component summary

Parameter	Robust group (n = 234)	Respiratory sarcopenia group (n = 135)	p value
Age (year), median (IQR)	77 (65-94)	78 (65-90)	0.290
Sex (female), n (%)	203 (86.8)	109 (80.7)	0.124
BMI (kg/m^2^), median (IQR)	21.4 (15.1-33.3)	21.9 (14.7-33.9)	0.244
Education history (year), median (IQR)	12 (6-21)	12 (9-26)	0.563
Medical history, n (%)			
Cancer	29 (12.4)	15 (11.1)	0.714
Heart disease	18 (7.7)	15 (11.1)	0.268
Stroke	10 (4.3)	2 (1.5)	0.145
Hypertension	76 (32.5)	40 (29.6)	0.570
Diabetes	17 (7.3)	8 (5.9)	0.622
Dyslipidemia	26 (11.1)	16 (11.9)	0.829
Fracture	6 (2.6)	4 (3)	0.820
Osteoporosis	36 (15.4)	21 (15.6)	0.965
Osteoarthritis	44 (18.8)	22 (16.3)	0.545
Dementia	0 (0)	0 (0)	-
Frailty, n (%)			
Prefrailty	87 (37.2)	61 (45.1)	0.077
Frailty	42 (17.9)	30 (22.2)	
Sarcopenia, n (%)			
Sarcopenia	6 (2.6)	4 (3)	0.916
Severe sarcopenia	4 (1.7)	3 (2.2)	
BIA			
SMI (kg/m^2^), median (IQR)	6.4 (4.6-9.7)	6.4 (5.4-8.6)	0.981
BF (kg), median (IQR)	14.4 (2.2-36.8)	15 (4.4-41.8)	0.147
FFM (kg), median (IQR)	35.2 (27.1-62)	35.6 (27.5-55.9)	0.106
PBF (%), mean ± SD	28.8 ± 8.1	29.6 ± 8	0.363
PhA (°), median (IQR)	4.6 (3.1-7.3)	4.5 (3.1-7.6)	0.104
Physical function			
Hand grip (kg), median (IQR)	22 (10.3-43.2)	21 (11.7-43.1)	0.039^*^
Gait speed (m/s), median (IQR)	1.4 (0.6-2.5)	1.3 (0.2-2.2)	0.006^*^
5Chair stand (seconds), median (IQR)	6.8 (3.6-17.6)	7.9 (3.1-20.2)	<0.001^*^
IPAQ, n (%)			
Low	67 (28.6)	63 (46.7)	0.002^†^
Moderate	112 (47.9)	46 (34.1)	
High	55 (23.5)	26 (19.3)	
Respiratory function			
MIP (cmH_2_O), median (IQR)	-48.1 (-111.8 to 15.6)	-27 (-67.3 to 9.3)	<0.001^*^
%MIP (%), median (IQR)	99.2 (33.6-282)	55.9 (18.6-79.6)	<0.001^*^
MEP (cmH_2_O), median (IQR)	51.7 (16.9-139.7)	35.8 (14.4-77.8)	<0.001^*^
%MEP (%), median (IQR)	83.5 (27.6-189.4)	57 (13.8-79.7)	<0.001^*^
Oral function			
ODK (times/s), median (IQR)			
Pa	6.6 (3.6-8)	6.2 (2.8-8.6)	0.001^*^
Ta	6.4 (3.8-8)	6.2 (3.4-8.8)	0.002^*^
Ka	6 (3.8-8)	5.8 (3.2-7.6)	0.002^*^
TP (kPa), median (IQR)	30.8 (13.4-54.3)	27.4 (8.6-54.3)	<0.001^*^
Cognitive function			
MoCA-J (score), median (IQR)	25 (14-30)	24 (13-30)	0.002^*^
QOL			
SF-8 PCS (score), median (IQR)	48.5 (21.3-63.7)	47.9 (25.5-57.3)	0.125
SF-8 MCS (score), median (IQR)	51.4 (26.6-62.8)	50 (31.8-61)	0.118

Factors associated with respiratory sarcopenia

Table 3 presents the results of the single regression analysis using the respiratory sarcopenia group as the dependent variable. The analysis identified decreased physical activity, reduced walking speed, prolonged time required for 5CS, and declines in oral and cognitive function as risk-related factors for respiratory sarcopenia. Based on these findings, the logistic regression analysis was conducted, and the results are shown in Table 4. Significant factors associated with respiratory sarcopenia included decreased grip strength, increased 5CS time, reduced moderate physical activity, decreased tongue pressure, and higher FFM. Notably, prolonged 5CS time and greater FFM were found to be more strongly associated with the onset of respiratory sarcopenia.

**Table 3:** Simple regression analysis results ^*^p value of <0.05 was considered statistically significant OR, odds ratio; CI, confidence interval; BMI, body mass index; SMI, skeletal muscle index; FFM, fat-free mass; PBF, percent body fat; PhA, phase angle; IPAQ, International Physical Activity Questionnaire; 5CS, 5 chair-stand; TP, tongue pressure; MoCA-J, Montreal Cognitive Assessment-Japanese

Variable	Β	OR	95% CI	p value
Age, year	0.021	1.021	0.984-1.059	0.265
Sex (female), n	-0.446	0.640	0.362-1.133	0.126
BMI, kg/m^2^	0.033	1.033	0.969-1.103	0.320
Sarcopenia, n				
Robust	-	-	-	0.916
Sarcopenia	0.154	1.167	0.323-4.211	0.814
Severe	0.272	1.312	0.289-5.957	0.725
SMI, kg/m^2^	0.051	1.052	0.766-1.446	0.752
FFM, kg	0.032	1.033	0.994-1.073	0.099
PBF, %	0.012	1.012	0.986-1.039	0.363
PhA, °	-0.184	0.832	0.555-1.248	0.374
IPAQ, n				
Low	-	-	-	0.002^*^
Moderate	-0.828	0.437	0.269-0.710	<0.001^*^
High	-0.688	0.503	0.282-0.898	0.020^*^
Hand grip, kg	-0.027	0.974	0.938-1.011	0.164
Gait speed, m/s	-1.217	0.296	0.137-0.341	0.002^*^
5CS, seconds	0.288	1.333	1.196-1.486	<0.001^*^
Pa, times/second	-0.41	0.664	0.523-0.842	<0.001^*^
Ta, times/second	-0.404	0.667	0.524-0.850	0.001^*^
Ka, times/second	-0.467	0.627	0.480-0.818	<0.001^*^
TP, kPa	-0.055	0.946	0.919-0.974	<0.001^*^
MoCA-J, score	-0.088	0.916	0.862-0.972	0.004^*^

**Table 4:** Logistic regression analysis results ^*^p value of <0.05 was considered statistically significant OR, odds ratio; CI, confidence interval; FFM, fat-free mass; PhA, phase angle; IPAQ, International Physical Activity Questionnaire; 5CS, 5 chair-stand; ODK, oral diadochokinesis; TP, tongue pressure

Variable	Β	OR	95% CI	p value
Sex (female), n	-0.779	0.459	0.115-1.826	0.269
Age, year	-0.023	0.978	0.933-1.024	0.342
Sarcopenia, n				
Robust	-	-	-	0.186
Sarcopenia	-0.637	0.529	0.109-2.570	0.430
Severe	-1.784	0.168	0.024-1.163	0.071
FFM, kg	0.100	1.106	1.009-1.211	0.031^*^
PhA, °	-0.283	0.753	0.452-1.256	0.277
IPAQ, n				
Low	-	-	-	0.061
Moderate	-0.656	0.519	0.301-0.894	0.018^*^
High	-0.300	0.741	0.389-1.410	0.741
Hand grip, kg	-0.109	0.896	0.833-0.965	0.004^*^
Gait speed, m/s	-0.141	0.869	0.321-2.352	0.782
5CS, seconds	0.196	1.216	1.067-1.386	0.003^*^
ODK (Ta), times/second	-0.249	0.779	0.584-1.040	0.091
TP, kPa	-0.043	0.958	0.928-0.989	0.009^*^

## Discussion

This study examined the prevalence and characteristics of respiratory sarcopenia and associated factors in community-dwelling older adults. The prevalence of Probable respiratory sarcopenia was 3.3%, and that of Possible respiratory sarcopenia was 33.3% among community-dwelling older adults. Individuals with respiratory sarcopenia exhibited significantly lower levels of physical function, physical activity, oral function, and cognitive function compared with the Robust group. Our findings revealed that physical function, physical activity, oral function, and FFM were significant factors for respiratory sarcopenia.

The strength of this study is that it is the first to comprehensively investigate the factors involved in respiratory sarcopenia, including physical function, oral function, cognitive function, and quality of life.

In a previous study, the proportion of people with reduced respiratory muscle strength was 54.7%, and the proportion of people with systemic sarcopenia and reduced respiratory muscle strength was 12% [4]. However, in our study, the values were lower. While a previous study used MIP to assess respiratory muscle strength [4], defining respiratory muscle weakness as being below the predicted value corrected for age and sex, our study defined respiratory muscle weakness as being below 80% of the predicted value, in accordance with the report by Miyazaki et al. [18]. This likely resulted in a small number of participants with respiratory muscle weakness. Moreover, our study followed the position paper and used cases of reduced respiratory muscle strength, including both expiratory and inspiratory muscles, to determine respiratory sarcopenia. This approach may have resulted in a stricter classification and a lower prevalence of respiratory sarcopenia compared with a previous study [4].

Respiratory sarcopenia is associated with significantly reduced physical function. From a previous study, respiratory muscle strength was associated with physical functions, such as grip strength, walking speed, and chair standing [4], similar to a previous study [4]. Grip strength is often used as a proxy for overall muscle strength [20]. Therefore, it is likely that grip strength was associated with respiratory muscle strength in this study. In a study on older adults requiring long-term care and support in Japan, reduced walking speed has been associated with impaired postural control function (role in maintaining postural and movement stability during walking) owing to reduced respiratory muscle strength [21]. It is possible that the reduced walking speed in the respiratory sarcopenia group was related to postural control. Reduced standing speed is also associated with postural control dysfunction in patients with chronic obstructive pulmonary disorder [22]. Therefore, we hypothesize that the 5CS speed was also reduced in the respiratory sarcopenia group. The association between grip strength and 5CS with respiratory sarcopenia is assumed to be due to a respiratory metabolic reflex, whereby reduced respiratory muscle strength causes dyspnea and physical activity limitation, resulting in limb skeletal muscle strength [10].

Significant differences in physical activity were found in our study, identified as factors associated with respiratory sarcopenia. Similar to a previous study, sedentary older adults experienced respiratory muscle weakness and dyspnea, causing physical inactivity and low activity in the United States [6]. For each physical activity category, the Robust group had a higher proportion of moderate physical activity, whereas the respiratory sarcopenia group had a higher proportion of low physical activity. Thus, older adults with respiratory sarcopenia may have activity limitations due to dyspnea, leading to inactivity and low physical activity. Additionally, the aforementioned respiratory metabolic reflex may be involved [10]. Although our study has a cross-sectional design, which limits the ability to establish causal relationships, physical activity may play a crucial role in the development or progression of respiratory sarcopenia.

Cognitive function was significantly lower in the respiratory sarcopenia group. Although previous studies have not reported a direct association between respiratory muscle strength and cognitive function, several have demonstrated associations between cognitive function and pulmonary function parameters, such as peak expiratory flow, forced vital capacity, and forced expiratory volume [23]. This study suggests that good respiratory function may help protect the brain from chronic hypoxia and ischemic injury. Chronic hypoxia has been linked to impaired ATP production, abnormal synthesis of neurotransmitters (e.g., acetylcholine), oxidative stress, and blood-brain barrier dysfunction, all of which have been suggested as possible contributors to the pathogenesis of Alzheimer’s disease. While not a direct explanation, our findings are considered to be in line with and supportive of this previous observation.

The respiratory sarcopenia group had significantly poorer oral function. The ODK assesses tongue and lip motor function, which is associated with swallowing function in community-dwelling older adults [24]. Tongue pressure is an indicator of swallowing muscle strength [25]. As safe swallowing requires coordination between breathing and swallowing [26], oral function may be related to respiratory sarcopenia. Tongue pressure is also a relevant factor in respiratory sarcopenia. Low tongue pressure can impede the formation and propagation of food clumps, leading to reduced oral intake and malnutrition [27]. A reduced tongue pressure can cause reduced oral intake and low nutritional status, associated with respiratory sarcopenia. However, the amount of FFM, considered an indicator of nutrition [28], differed from the hypothesized results.

Comparing the mean FFM of each group with that of the Japanese population [29] revealed that women in our study had lower FFM values. This suggests that our study participants may have had a lower overall nutritional status compared with the Japanese population. No significant differences in FFM were observed between the respiratory sarcopenia and Robust groups. It is considered that the decline in oral function may have led to reduced food intake, which in turn could be related to respiratory sarcopenia. However, since food intake was not measured in this study, this association cannot be confirmed, and the association between poor nutritional status and respiratory sarcopenia cannot be clarified.

The findings of this study suggest that respiratory sarcopenia is associated with physical function, physical activity, oral function, and cognitive function in community-dwelling older adults. This highlights the importance of screening for respiratory sarcopenia in both clinical and community settings, as early identification may contribute to the maintenance of independence and the prevention of sarcopenia in older individuals.

The limitations of this study include the predominance of female participants, which introduces a potential gender bias. Additionally, respiratory muscle mass was not measured, which may have limited the accuracy of the diagnosis of respiratory sarcopenia. Moreover, respiratory function tests were not conducted, making it possible that individuals with low respiratory muscle strength due to functional impairment were misclassified as having possible sarcopenia. While the prevalence of sarcopenia among community-dwelling older adults in Japan has been reported to be 9.9% [30], the prevalence in this study was only 4%, suggesting that the prevalence of respiratory sarcopenia may have been underestimated. Furthermore, this study involved a comprehensive functional assessment including not only respiratory muscle strength, but also physical, cognitive, and psychosocial functions. Since participation required individuals to walk independently to the venue, only those with a certain level of mobility were included. Although participants were considered to have independent ADL, the diagnostic criteria for respiratory sarcopenia in the position paper do not require ADL independence, which may have led to underestimation of its prevalence. Finally, given the cross-sectional design of this study, it is not possible to establish causal relationships between respiratory sarcopenia and its associated factors. Therefore, longitudinal studies are needed in the future.

## Conclusions

The prevalence of Probable respiratory sarcopenia and Possible respiratory sarcopenia among community-dwelling older adults was 3.3% and 33.3%, respectively. Respiratory sarcopenia was characterized by significantly lower physical function (grip strength, gait speed, and 5CS), physical activity, oral function (ODK and tongue pressure), and cognitive function. Furthermore, grip strength, 5CS score, moderate physical activity, and tongue pressure were identified as significant factors associated with respiratory sarcopenia, suggesting that it requires a comprehensive evaluation including physical function, physical activity, oral function, and cognitive function. This highlights the importance of screening for respiratory sarcopenia in both clinical and community settings, as early identification may contribute to the maintenance of independence and the prevention of sarcopenia in older individuals.
